# Ocular ultrasound versus MRI in the detection of extrascleral extension in a patient with choroidal melanoma

**DOI:** 10.1186/s12886-018-0990-0

**Published:** 2018-12-12

**Authors:** Bradley H. Jacobsen, Christopher Ricks, Roger P. Harrie

**Affiliations:** Department of Ophthalmology, University of Utah, Salt Lake City, UT USA

**Keywords:** Ocular ultrasound, Uveal melanoma, Extrascleral extension, MRI, Metastatic uveal melanoma, Choroidal melanoma

## Abstract

**Background:**

To describe the superiority of ocular ultrasound in the diagnostic management of extrascleral extension in choroidal melanoma.

**Case presentation:**

We present a case of a 94-year-old male with choroidal melanoma of the right eye imaged with MRI and ocular ultrasound to aid in the detection of extrascleral extension.

**Conclusions:**

With advancement in technology and new imaging modalities emerging, it can become difficult to determine the best diagnostic approach for patients. We believe that ocular ultrasound remains the superior imaging modality in detection of extrascleral extension in choroidal melanoma.

## Backgroud

Uveal melanoma is the most common intraocular malignancy and represents 5% of all melanoma diagnoses in the United States [1, 2]. Currently ocular ultrasound is the imaging modality of choice in monitoring the progression of uveal melanoma and detecting extrascleral extension. However, with advancement in technology MRI is proving to be a valuable tool in the diagnosis of extrascleral extension.

### Case report

A 94-year-old male with a past ocular history of age-related macular degeneration (AMD) in both eyes presented to the ophthalmology clinic for a routine dilated fundus exam (DFE). On exam his Snellen visual acuity was 20/100 OD and count fingers (CF) OS. Exam findings were significant for end-stage AMD in the left eye and subretinal hemorrhage in the right eye. He was referred for ocular ultrasound and found to have subretinal hemorrhage secondary to progression of his exudative macular degeneration. Anti-VEGF treatment was initiated and continued for several months. The patient was then found to have a choroidal lesion that measured 14.5 × 14.6 mm in basal dimension with a thickness of 6.4 mm. There was low to medium reflectivity, moderate irregularity and trace spontaneous vascularity. These findings were consistent with a clinical diagnosis of choroidal melanoma.

**Fig. 1:**
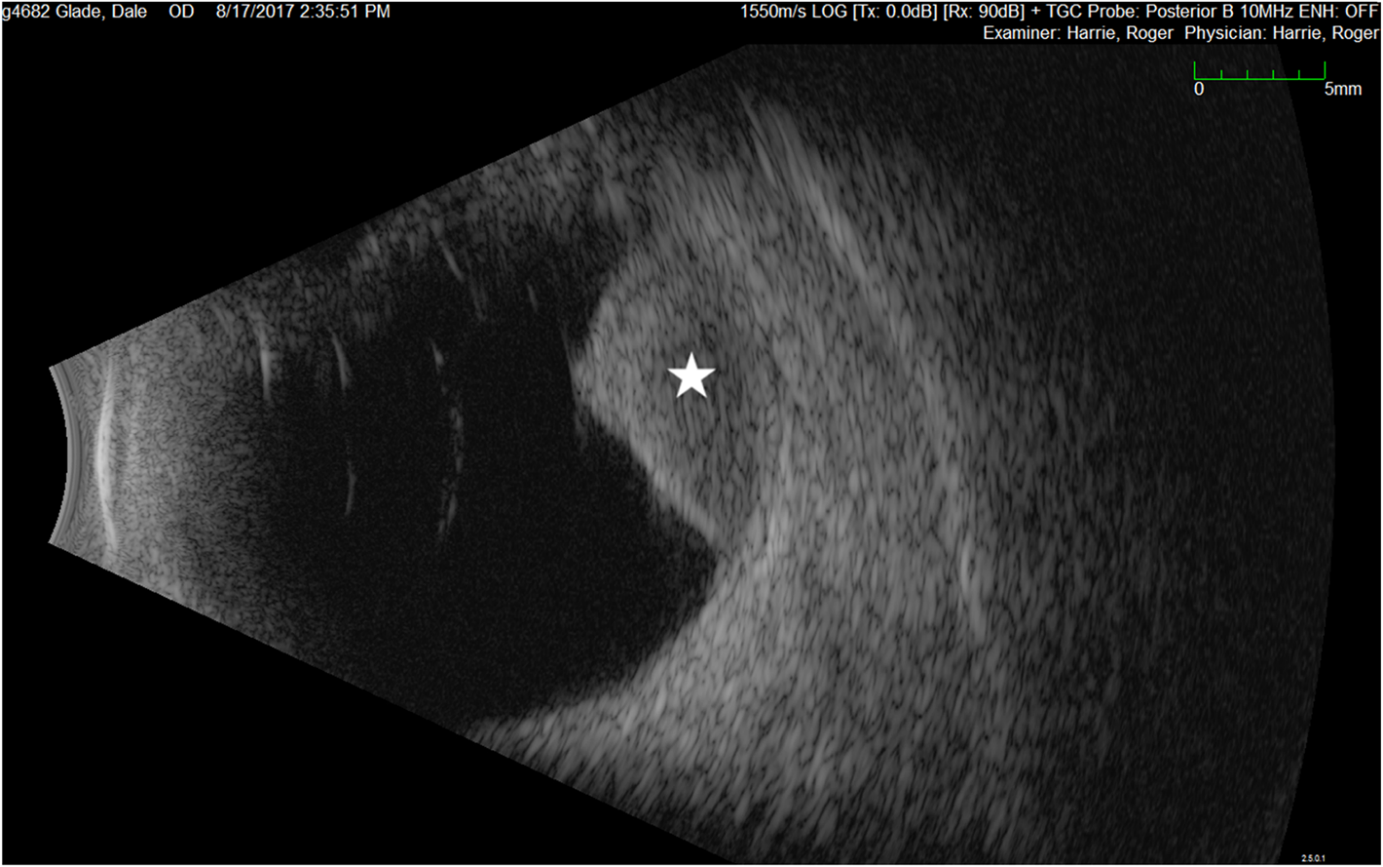
B-Scan Ultrasound of patients right eye showing the classic collar button (mushroom) shape of tumor (white star) without evidence of extrascleral extension

**Fig. 2:**
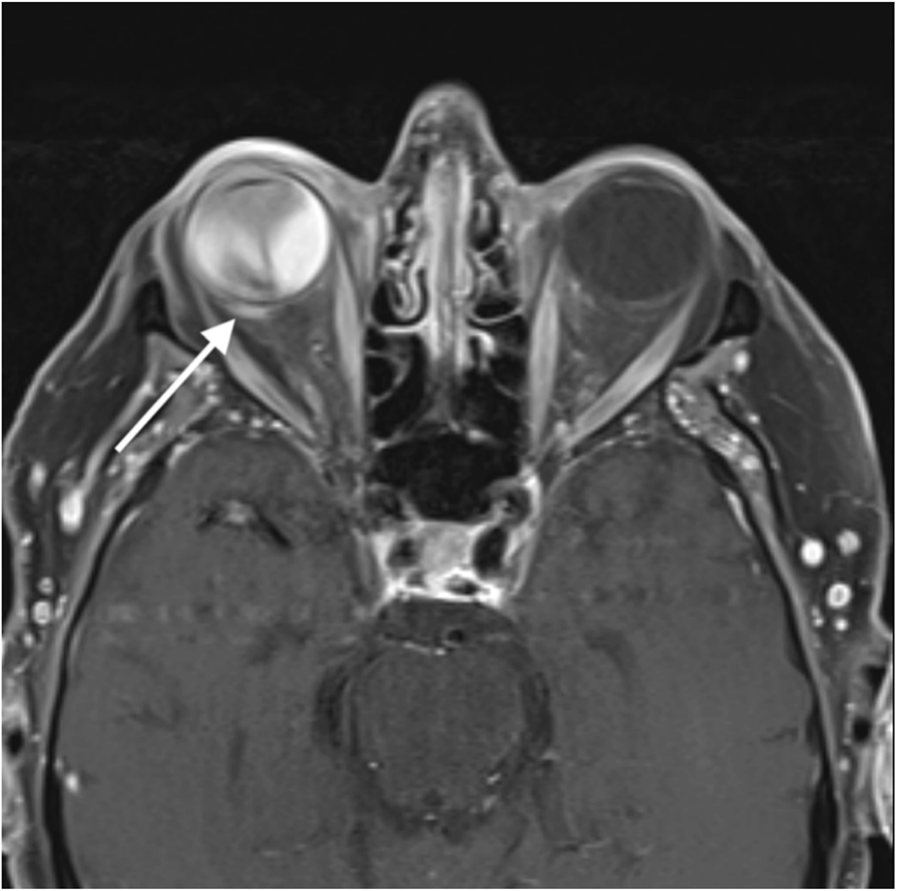
Axial post-contrast MRI orbits showing what appears to be contrast enhancement extending into the immediately adjacent retro-bulbar fat (white arrow), suspicious for extrascleral invasion

The patient was referred for a liver ultrasound, which showed questionable focal lesions within the liver, and further evaluation with CT abdomen and pelvis was recommended. Given the liver ultrasound findings the patient was re-evaluated with ocular ultrasound, which did not show evidence of extrascleral extension (Fig. 1). The patient underwent staging CT chest, abdomen and pelvis that were negative for metastatic disease. An MRI of the orbits with contrast showed a subcentimeter region of abnormal contrast enhancement extending into the immediately adjacent retro-bulbar fat, suspicious for scleral invasion and small extrascleral lesional extension (Fig. 2).

**Fig. 3:**
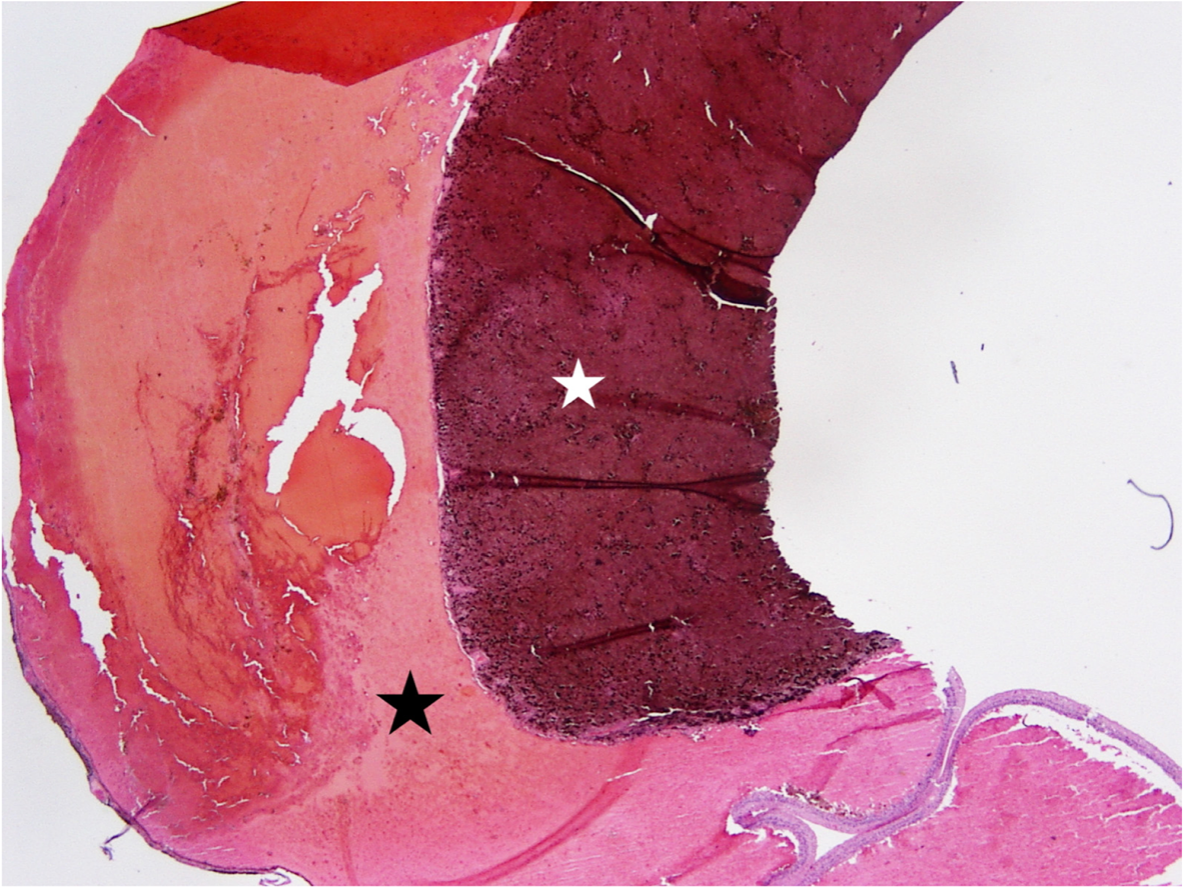
H&E stain of right eye with intact sclera (black star) and no obvious extrascleral extension of choroidal melanoma (white star) posteriorly

The patient presented to the ophthalmology walk-in clinic several days after the MRI with complaints of right eye pain that he described as “monotonous friction” like pain. Exam findings were significant for visual acuity no light perception (NLP) OD, an intraocular pressure of 28 OD, diffuse hemorrhage in the anterior chamber and no view into the posterior pole secondary to vitreous hemorrhage. B-scan ocular ultrasound performed during that visit was consistent with hemorrhagic choroidal detachments with diffuse vitreous hemorrhage. Given the presence of a choroidal melanoma, questionable extrascleral extension and a painful eye, patient and providers decided to pursue enucleation. The patient underwent enucleation OD and the specimen was sent for analysis. Final pathology revealed malignant melanoma of the choroid (spindle B-type) with intact sclera and no obvious extrascleral extension posteriorly (Fig. 3).

## Discussion

Uveal melanoma is the most common intraocular malignancy in adults and represents 5% of all melanoma diagnoses in the United States [1, 2]. Detection and monitoring of uveal melanoma is important, as survival correlates with tumor size [1]. Although uveal melanomas can occasionally be associated with metamorphopsia, retinal detachments and photopsias, many are asymptomatic and found on routine ophthalmic exam [3]. Clearly indirect ophthalmoscopy is the most important initial examination technique in the diagnosis of uveal melanoma. However, the use of ocular ultrasonography and MRI/CT are important adjuncts in the detection of intrascleral invasion and extrascleral extension of uveal melanoma.

It is well known that ocular ultrasonography is the most important ancillary tool for evaluating and tracking progression of uveal melanomas. In addition, it is often the imaging modality of choice in detecting extrascleral extension [4]. However, there are new studies emerging that challenge this idea, which suggest that other imaging modalities such as MRI are superior to ocular ultrasound in the detection of extrascleral extension.

Récsán, et al. studied the value of MRI for the detection of extrascleral extension of uveal melanoma in 12 patients. In this study MRI had a sensitivity and specificity of 100 and 89%, respectively, for detection of extrascleral extension [5]. In another study Burris, et al. looked at 16 eyes with histopathological evidence of extrascleral extension. They found that only 8 of the 16 cases had detection of extrascleral extension preoperatively with the use of ocular ultrasound [6]. Scott, et al. challenged this and directly compared the sensitivity of ocular ultrasound vs MRI/CT in the detection of extraocular extension. Of the 10 patients who underwent ocular ultrasound, extraocular tumor extension was demonstrated in 100% of patients. This was superior to MRI or CT scan, which only detected extraocular tumor extension in 29 and 0% of patients’ respectively [7].

Our patient underwent both MRI and ocular ultrasound in an attempt to detect extrascleral extension. Ocular ultrasound proved to be superior to MRI in our patient. The findings on MRI that indicated possible extraocular extension were reviewed with the neuro-radiology department after the pathology showed no extension. In hind-sight the concerning MRI findings were thought, in order of likelihood, to be caused by non-specific inflammation, a vascular formation (such as a feeder vessel) or motion artifact.

These findings cannot be generalized to every patient and the evaluation and treatment should be planned on a case-by-case basis. Ultrasound should be performed by an experienced provider in all patients with uveal melanoma. Although every patient is unique in his or her presentation, and resources may be limited, we feel that ocular ultrasound should be the imaging modality of choice when looking for extrascleral extension in uveal melanoma.

## Abbreviations

CF: Count fingers
DFE: Dilated fundus exam
NLP: No light perception
OD: Right eye
OS: Left eye
VEGF: Vascular endothelial growth factor
